# Supplementary material for: Complexome profiling of the Chlamydomonas psb28 mutant reveals TEF5 as an early PSII assembly factor
Source: Plant Cell. 2025 Mar 17;37(6):koaf055. doi: 10.1093/plcell/koaf055 (PMC12164590; doi:10.1093/plcell/koaf055)
Supplement: koaf055_Supplementary_Data [file koaf055_supplementary_data.zip › psb28_interactive.zip!/plots/intensity/qualityPlots/peptides/CHLG_Cre06.g294750.t1.2.html]

### Cre06.g294750.t1.2

Trivial name: CHLG  
SeqCoverage [median]: 17.50  
Mass: 41.45 kDa  
  
 

| Sequence | MissedCleavage | Mass [Da] | wtIntensity | muIntensity | Score |
| --- | --- | --- | --- | --- | --- |
| EIDAINEPYRPIPSGR | 1 | 1825.93 | 20339.29 | 65068.65 | 162.37 |
| GAALETDIWK | 0 | 1102.57 | 14055.40 | 40188.25 | 137.13 |
| QMGLQSLPVAFGVDTAK | 0 | 1760.91 | 1957.20 | 4119.33 | 146.79 |
| QMLGMK | 0 | 706.35 | 11550.86 | 9393.84 | 110.54 |
| SIEGDRQMGLQSLPVAFGVDTAK | 1 | 2418.22 | 0.00 | 4228.98 | 69.72 |
| YFLPDPIANDVK | 0 | 1390.71 | 18653.64 | 32064.82 | 144.65 |
|  |  |  | 66556.4 | 155063.9 |  |
